# A Hyperbranched Polyol Process for Designing and Manufacturing Nontoxic Cobalt Nanocomposite

**DOI:** 10.3390/polym15153248

**Published:** 2023-07-30

**Authors:** Anastasia Burmatova, Artur Khannanov, Alexander Gerasimov, Klara Ignateva, Elena Khaldeeva, Arina Gorovaia, Airat Kiiamov, Vladimir Evtugyn, Marianna Kutyreva

**Affiliations:** 1 A.M. Butlerov Chemical Institute, Kazan Federal University, 18 Kremlyovskaya Str., 420008 Kazan, Russia; nastyaburmatova15@gmail.com (A.B.); alexander.gerasimov@kpfu.ru (A.G.); toklara@yandex.ru (K.I.); e_khaldeeva@mail.ru (E.K.); gorovayaarina5@gmail.com (A.G.); vevtugyn@gmail.com (V.E.); mkutyreva@mail.ru (M.K.); 2 Kazan Research Institute of Epidemiology and Microbiology, 67 Bolshaya Krasnaya Str., 420015 Kazan, Russia; 3 Quantum Simulators Lab, Institute of Physics, Kazan Federal University, Kremlevskaya Str. 18, 420008 Kazan, Russia; airatphd@gmail.com

**Keywords:** hyperbranched polyesters, nanocomposite synthesis, cobalt nanoparticles, synthetic approach, antimycotic activity

## Abstract

A method for the design and synthesis of a metallopolymer composite (CoNP) based on cobalt nanoparticles using the hyperbranched polyol process was developed. It was shown that hyperbranched polyester polyols in a melted state can be both a reducing agent and a stabilizer of metal nanoparticles at the same time. The mechanism of oxidation of hyperbranched polyol was studied using diffuse reflectance IR spectroscopy. The process of oxidation of OH groups in G4-OH started from 90 °C and finished with the oxidation of aldehyde groups. The composition and properties of nanomaterials were determined with FT-IR and UV-Vis spectroscopy, Nanoparticle Tracking Analysis (NTA), thermogravimetric analysis (TG), powder X-ray diffraction (XRD), NMR relaxation, and in vitro biological tests. The cobalt-containing nanocomposite (CoNP) had a high colloidal stability and contained spheroid polymer aggregates with a diameter of 35–50 nm with immobilized cobalt nanoparticles of 5–7 nm. The values of R_2_ and R_1_ according to the NMR relaxation method for CoNPs were 6.77 mM·ms^−1^ × 10^−5^ and 4.14 mM·ms^−1^ × 10^−5^ for, respectively. The ratio R_2_/R_1_ = 0.61 defines the cobalt-containing nanocomposite as a *T*_1_ contrast agent. The synthesized CoNPs were nonhemotoxic (HC_50_ > 8 g/mL) multifunctional reagents and exhibited the properties of synthetic modulators of the enzymatic activity of chymosin aspartic proteinase and exhibited antimycotic activity against *Aspergillus fumigatus*. The results of the study show the unique prospects of the developed two-component method of the hyperbranched polyol process for the creation of colloidal multifunctional metal–polymer nanocomposites for theranostics.

## 1. Introduction

The creation of nanostructured composite materials based on metal nanoparticles is currently both a fundamental and practically significant interdisciplinary direction, which today determines the development of advanced technologies. The use of metal-containing nanomaterials in biomedicine and pharmacology opens ways to improve the bioavailability and efficacy of drugs, allows for the optimization of therapeutic protocols for disease treatment, and determines the strategy for creating new pharmaceutical substances to address antibiotic resistance and new chemical nanosystems to develop alternative treatments for socially significant diseases [1,2]. In addition, composites with immobilized nanoparticles of biophilic 3Dtransition metals are unique objects for the creation of multifunctional theranostic agents [3,4,5,6]. Magnetic or photophysical activity in combination with biospecificity with respect to enzymes or cellular structures allows them to be used both as diagnostic agents [7,8,9,10,11,12,13] and as therapeutic agents or vectors [14,15].

The most important functional properties of metal-containing composite nanomaterials used for these purposes, such as magnetic activity, photophysical properties, biological activity, and toxicity, are determined primarily by the composition and morphology of the material particles. In turn, these characteristics are controlled by the choice of components and synthetic approach. 

The polyol method is one of the most promising and ecofriendly approaches to the one-step synthesis of metal nanoparticles with a controlled shape and size [16,17,18,19]. Two reagents are central to this method. The first is the precursor of the metal nanophase (usually an inorganic salt). The second is a polyol. Linear low-molecular-weight and high-molecular-weight polyhydric alcohols, such as ethylene glycol [20,21], propylene glycol [22], and glycerin [23], are often used as a reducing agent in the polyol process. These reagents are most successful for the one-pot preparation of polymer-immobilized nanoparticles of the noble metals silver [24,25] and gold [26,27]. As a rule, the presence of co-reducing agents (as the third reagent) is required for the synthesis of 3D transition metal nanoparticles including magnetically active cobalt and iron nanocomposites [28,29,30,31]. In addition, a fourth compound—a stabilizer of the 3d metal nanophase—is always in the reaction system. These are most often alkylamines [32], polymers [33,34], surfactants [19], and simple anions [35,36]. The multicomponent composition of the reaction mixture complicates synthetic procedures, leads to side processes formed by-products, pollutes the nanoparticle’s surface, and induces their aggregation. A slight change in the sequence of the addition of reagents or their concentration significantly affects the morphology and functional properties of the metal nanoparticles. Therefore, the search for new approaches and reagents for the polyol synthesis of metal nanoparticles and metal composites is an important and practical task in the field of nanomaterial chemistry.

The use of hyperbranched dendritic-like polyols can become a new approach in the development of a one-pot polyol method for the successful synthesis of the nanoparticles of transition metals, including magnetically active ones. The significant amount and high density of hydroxyl groups in the peripheral layer of the polymer can ensure its success as a reducer of metal nanoparticles. In turn, the three-dimensional architecture of hyperbranched polymers is characterized by the presence of a spatially unloaded core [37,38] in combination with a dense functional shell. This defines hyperbranched polyols as excellent metal nanophase stabilizers. The variability in stabilization mechanisms by hyperbranched polymers [39] determines the morphology of composite nanoparticles, both noble [40] and transition [41,42] metals. 

Previously, in our studies, it was shown that hyperbranched polyester polyols are good stabilizers in the formation of composite nanoparticles of a number of biophilic metals (copper [40], iron [43], and cobalt [41]) via the chemical recovery method, using hydrazine hydrate or sodium borohydride as active reducing agents. Among the synthesized nanomaterials, superparamagnetic polyol-stabilized cobalt nanoparticles are of particular interest as they can be used as multifunctional nanocomposites for biomedicine, advanced technologies, and theranostics. Hyperbranched polyester polyols can act not only as a stabilizer, but also have prospects for targeted use [44,45]. However, the cobalt-containing nanocomposites obtained previously required thorough purification from by-products and had the greatest stability in powder form. Nanomaterials are the most important for medical applications, with their high colloidal stability. The possibilities of hyperbranched polyester polyols as a reducing agent in the polyol method for the synthesis of colloidal composite metal nanoparticles have not been previously studied. Thus, the aim of this work is to develop an approach to the synthesis of colloidal cobalt nanoparticles through a two-compound hyperbranched polyol process and to evaluate the morphology, properties, and functional activity of a cobalt-containing nanocomposite. 

## 2. Materials and Methods

### 2.1. Materials 

For the synthesis of cobalt-containing nanocomposites (CoNPs), we used hyperbranched bis-MPA polyester polyol, generation 4-G4-OH (Sigma-Aldrich, St. Louis, MO, USA, CAS: 326794-48-3, lot. 369029, 64 hydroxyl groups, M_r_ = 7323.32 g·mol^−1^, hydroxyl number 486 mg KOH × g^−1^), and salt CoCl_2_·6H_2_O (99%, Alfa Aesar, Haverhill, MA, USA) as a cobalt precursor. 

To assess the content of cobalt in the composition of the nanocomposite by titration, we used a 1 M aqueous solution of HCl in methanol (1:1) for sample preparation and EDTA disodium salt (Sigma-Aldrich) and the murexide indicator (Sigma-Aldrich) for titration.

To study the hemolytic activity of CoNPs, blood from healthy donors was obtained from the blood donor center in Kazan and was anticoagulated with 3% sodium citrate.

The evaluation of proteolytic activity in the presence of CoNPs was carried out for the enzyme–substrate system: chymosin (CHY-MAX Powder Extra NB, Chr. Hansen, Denmark., CAS 9001-98-3 Rennet Powder 20,000 u/g)–bovine hemoglobin (SKU: 212392, BBL^TM^, USA). Chymosin and hemoglobin solutions were prepared in PBS with pH = 3.58, stored at 4–5 °C, and used within 24 h.

The antimycotic activity of CoNPs was evaluated on cell cultures of *Candida albicans* ATCC 10201 and *Aspergillus fumigatus* F-753 provided by the Laboratory of Fungal Allergens of the Kazan Research Institute of Epidemiology and Microbiology of the Russian Federation. 

The solvents for the synthesis and isolation of cobalt-containing nanocomposites were acetone, DMSO, and petroleum ether purified according to the standard procedures. CoNP samples for TEM were prepared in methanol. In experiments to study the properties of the CoNPs, we used deionized water (Ω = 18.2 MΩ·cm at 25 °C, χ = 0.055 µS/cm, particle number of 0.22 µm/mL < 1) and phosphate-buffered saline (PBS) with pH = 7.4.

### 2.2. Equipment

The FT-IR spectra were recorded on a Spectrum 400 (PerkinElmer, Waltham, MA, USA) ATR-FT-IR spectrometer. The FT-IR spectra from 4000 to 400 cm^−1^ were considered in this analysis. The spectra were measured with 1 cm^−1^ resolution and 64-scan coaddition. 

To establish the mechanism of oxidation of polyester polyol G4-OH, FT-IR spectra were recorded on a Spectrum 400 Fourier spectrometer (PerkinElmer) with a diffuse reflectance attachment with a compact temperature controller “PIKE technologies” in the temperature range 35–210 °C, 4000–400 cm^−1^ (1 cm^−1^ resolution, 16-scan accumulation, and shooting range 4000–400 cm^−1^).

The electronic absorption (UV-Vis) spectra were recorded on a Lambda 750 (PerkinElmer) in the wavelength range of 200−1000 nm at T = 25 ± 0.01 °C, using a temperature-maintaining system including a cell holder flow thermostat “Julabo MB-5A” and a Peltier PTP-1 thermostat. Quartz cells with a thickness of 1 cm were used for the measurements. The measurement accuracy for absorbance (A) was ±1%.

The colloidal properties were studied via the Nanoparticle Tracking Analysis (NTA) method using a NanoSight LM-10 instrument (Malvern Panalytical, Malvern, England). The CMOS camera C11440-50B with an image capture sensor FL-280 from Hamamatsu Photonics (Hamamatsu, Japan) was used as a detector. The measurements were carried out in a special cuvette for aqueous solutions, equipped with a laser with a wavelength of 405 nm (CD version S/N 2990491); the O-ring was made of Kalrez material. The temperature in the chamber was determined using a contact thermometer OMEGA HH804 (Engineering Inc., Norwalk, CT, USA) for all measurements. For the spectra fitting, the OriginPro program package was used; the Gauss function was used throughout [43].

Thermogravimetric analysis (TG) was performed using a thermal analyzer STA 449 F1 Jupiter (Netzsch, Selb, Germany) with a temperature rate of 10 K·min^−1^ in an argon atmosphere and a total flow rate of 75 mL·min^−1^. The analysis was performed in a temperature range of 40–600 °C in an Al crucible with a volume of 40 µL that had lids with 3 holes, each of 0.5 mm in diameter. For the experiment, a sample of colloidally stable particles was precipitated with water and washed successively with acetone and diethyl ether.

Powder X-ray diffraction (XRD) was achieved with a Bruker D8 Advance apparatus with Cu Kα irradiation (λ = 1.5418 Å) in the Bragg–Brentano geometry. The rate was 0.18°/min; the range of 2θ angle was from 7° to 100°; and the step was 0.015°. For the experiment, a sample of colloidally stable particles was precipitated with water and washed successively with acetone and diethyl ether.

Proton relaxation times (spin–spin (transverse) *T*_2_ and spin–lattice (longitudinal) *T*_1_) were measured using a Minispec MQ20 pulsed NMR relaxometer from Bruker with an operational frequency of 19.65 MHz by applying the standard Carr–Purcell pulse sequence modified by Meiboom and Gill, with a measuring accuracy error smaller than 3%. The experimentally measured relaxation times (*T*_2_)_*obs*_ were inverted into the relaxation rates (1/*T*_2_)_*obs*_. The relaxation rate is the sum of the two main contributions: the relaxation of protons in bulk *n*-hexane (1/*T*_2_)_*d*_ (diamagnetic component) and the relaxation of the protons around the paramagnetic ion (1/*T*_2_)_*p*_ (paramagnetic component) [46]:

$$({\frac{1}{T_{1,2}\;})}_{obs}={\left(\frac{1}{T_{1,2}\;}\right)}_{p}+{\left(\frac{1}{T_{1,2}\;}\right)}_{d}$$

Transmission electron microscope (TEM) imaging was carried out in a Hitachi HT7700 (Tokyo, Japan) Excellence transmission electron microscope at an accelerating voltage of 100 kV in the TEM mode. The size and shape of the synthesized nanoparticles were estimated using ImageJ.

The ultrasonic treatment of dispersions for sorption studies was carried out in a sapphire ultrasonic bath (T = 25 °C; operating frequency, 35 kHz).

### 2.3. Synthesis of Colloidal-Cobalt-Containing Nanocomposite CoNPs Using the Hyperbranched Polyol Process

Polyester polyol G4-OH (5.0349 g) was dissolved in 8 mL of DMSO and was mixed at room temperature until the polymer was completely dissolved. Then, 0.0207 g CoCl_2_·6H_2_O (ν_Co(II)_/ν_OHpolyester polyol_ = 1:500) was added to the reaction mixture, which was then heated at 40 °C until the salt was completely dissolved and was left at room temperature for 2 days for self-organization. Then, the reaction mixture was heated to 240 °C in steps of 10 °C with constant stirring. The formation of a colloidal stable cobalt-containing nanocomposite (CoNPs-1 or CoNPs-2) was accompanied by a change in the color of the reaction mixture from blue to brown. Solid NaOH (ν_NaOH_/ν_Co(II)_ = 1:10) was added additionally to the reaction mixture with constant stirring to synthesize CoNPs-2. Water and a combination of acetone and petroleum ether were used to treat the reaction mixture in order to extract CoNPs.

CoNPs-1. IR spectrum, ν, cm^−1^: 3447 (OH_bonded_), 2978 (CH_3_), 2943 (CH_2_), 2885 (CH_2_), 1733 (C=O_ester._), 1713 (C=O_aldehyde_), 1470 [δ (CH_3_)], 1397 [δ (CH_2_)], 1373 [δ (CH_2_)], 1298 [δ (OH)], 1239 (C-O_ester._), 1125 (O-C_ester_), 1048 (O–C_hyd_), 1090 (Co-O-H), 1008 (C–O), 579 (Co^3+^_Co3O4_), 668 (Co^2+^_CoO_).
UV-Vis spectrum, λ_max_, nm: 250 (Co^0^_cluster_), 273 (Co^0^), 396 (Co_3_O_4_).
CoNPs-2. IR spectrum, ν, cm^−1^: 3394 (OH_bonded_), 2986 (CH_3_), 2943 (CH_2_), 2838 (CH_2_), 1727 (C=O_ester._), 1707 (C=O_aldehyde_), 1470 [δ (CH_3_)], 1447 [δ (CH_2_)], 1369 [δ (CH_2_)], 1307 [δ (OH)], 1232 (C-O_ester._), 1127 (O-C_ester_), 1048 (O–C_hyd_), 1028 (Co-O-H), 1013 (C–O), 570 (Co^3+^ _Co3O4_), 656 (Co^2+^_CoO_).
UV-Vis spectrum, λ_max_, nm: 276 (Co^0^), 390 (Co_3_O_4_).

### 2.4. Sorption Studies 

The sorption of Co^2+^ ions by polyester polyol G4-OH matrix was carried out in static conditions in DMSO. A series of solutions were prepared with a constant concentration of G4-OH (0.125 g·mL^−1^) and a variable concentration of CoCl_2_⋅6H_2_O (0.05–0.8 mol·L^−1^) in DMSO. Then, the mixtures were treated with ultrasound for 1 h and incubated at room temperature for 24 h. The resulting high-molecular associate [Co^2+^-G4-OH] was separated using centrifugation (v = 10,000 rpm, t = 5 min) and the free concentration of the Co^2+^ ion in the solution was determined with UV–Vis spectrophotometry according to the calibration graph at λ = 530 nm:

*A*_530_ = −(0.15 ± 0.01) + (12.1 ± 0.4) × *c_Co(II_*_)_, *R* = 0.9979

G4-OH intrinsic absorption was taken into account in the baseline. In parallel, control experiments were carried out with CoCl_2_·6H_2_O solutions at the same concentrations but without polymer H40. The sorption isotherm was plotted in the coordinates $c_{Co^{2+}}^{S}$ from $c_{Co^{2+}}^{0}$, where $c_{Co^{2+}}^{S}$ is the sorbed concentration of Co^2+^ and $c_{Co^{2+}}^{0}$ is the initial concentration of Co^2+^ in the solution.

### 2.5. Estimation of Cobalt Concentration in Nanocomposite CoNPs 

The cobalt concentration in the nanocomposite CoNPs was determined with complexometric titration. The weighed CoNPs (0.001 g) were dissolved in a mixture of 1 M aqueous solution of HCl in methanol (1:1) for ashing Co^0^ nanoparticles. An aliquot of the sample was then titrated with EDTA disodium salt solution (*C_EDTA_* = 0.1 M) with the murexide indicator until the yellow color changed sharply to violet. Determination was carried out at least three times. The concentration of cobalt ions was calculated with the equation:

$$C_{Co^{2+}}=\frac{V_{EDTA}\times C_{EDTA}}{V_{aliquot}\left(Co^{2+}\right)}$$

### 2.6. Hemolysis Assay 

A hemolysis assay was performed according to the Klajnert method [47]. Blood erythrocytes were separated from the plasma and leukocytes via centrifugation (5000× *g*, 5 min) at 4 °C and washed three times with phosphate-buffered saline (PBS). They were used immediately after isolation. The red blood cells (RBCs) were suspended in polymer solutions in PBS at a hematocrit of 1% and incubated for 0.5 h at a temperature of 20 °C to study the effects of the nanocomposites on hemolysis. The incubated suspensions were centrifuged at 1000× *g* for 5 min. For comparison, erythrocytes were treated with bidistilled water for 100% hemolysis. 

The hemolysis (%) was determined from the released hemoglobin in the supernatants and measured spectrophotometrically by absorbance at 540 nm

$$\mathrm{Hemolysis}\;\left[\%\right]={\frac{A-A_{-}}{A_{+}-A}}_{-}\times 100\%$$

where *A* is the optical density of the RBCs incubated with CoNPs, *A_−_* is the optical density of the CoNPs in PBS, and *A_+_* is the optical density of the RBCs in water (100% hemolysis). The nanocomposites themselves contributed no more than 0.1% of the absorbance at 540 nm. The results are expressed as mean ± standard deviation, n = 5. The value of HC_50_ corresponded to the concentration of CoNPs, at which 50% hemolysis of erythrocytes was observed [43].

### 2.7. Enzymatic Activity Studies 

The properties of CoNPs as a synthetic modulator of enzymatic activity were evaluated using the chymosin *(enzyme)*–hemoglobin *(substrate)* system via UV-Vis spectrophotometry. To determine the proteinase activity, we prepared solutions of chymosin (2 mg·mL^−1^) and hemoglobin-Hb (2 mg·mL^−1^) in PBS medium (pH = 3.58). Then, 0.1 mL of chymosin was added to a mixture containing 0.5 mL of Hb with and without CoNPs (0.001–10 mg·mL^−1^). The total volume of the sample was 1 mL; the chymosin concentration in the sample was 0.2 mg·mL^−1^, and the hemoglobin concentration in sample t was 1 mg·mL^−1^. After mixing and incubation at T = 50.0 ± 0.01 °C for 30 min, the samples were centrifuged at 10,000 rpm for 20 min. The supernatant was transferred into a quartz spectrophotometric cuvette and the electronic absorption spectrum was recorded in the wavelength range of 250–500 nm.

The concentration of Hb (c_Hb_ mg·mL^−1^) after proteolysis was estimated according to the calibration graph:

*A*_280_ = −(0.017 ± 0.001) + (0.0017 ± 0.0002) × *c_Hb_*, *R* = 0.9996

The contribution of the enzyme’s absorption was taken into account in the baseline. Each definition was repeated at least three times. 

The activity of chymosin (*A_E_*, mg·mL^−1^·min^−1^) was determined by the change in the rate of the catalytic reaction as compared to the noncatalytic one. The reaction rate was indicated as the change in the substrate concentration (mg·mL^−1^) per unit of time (min):

$$A_{E}\;\left[\mathrm{mg}\cdot {\mathrm{mL}}^{-1}\cdot \;\min{\;}^{-1}\right]=\frac{C_{Hb}^{0}-C_{Hb}^{}}{t},$$

where $C_{Hb}^{0}\;$ is the initial concentration of hemoglobin, and $C_{Hb}^{}$ is the concentration of the hemoglobin solution after proteolysis. 

The dependences of *A_E_* (*%*) *= f*(*lgC_CoNPs_*) were built according to the experimental data. Chymosin activity in the absence of cobalt-containing nanocomposite CoNPs was taken as $A_{E^{0}}$ or 100%. To assess the modulatory properties of CoNPs, the values of IC_50_ (half-maximal inhibition concentration) and AC_50_ (half-maximal activation concentration) were calculated.

### 2.8. Antimycotic Study

The antimycotic activity of the cobalt-containing nanocomposite CoNPs was evaluated at the Kazan Research Institute of Epidemiology and Microbiology on crops *Aspergillus fumigatus* F-753 and *Candida albicans* ATCC 10201. Studies of antimycotic activity of the CoNPs were carried out using a disk diffusion test on modified Saburo agar. Plants of test cultures (spore suspension) were applied at a rate of 1 million CFU/cup. Solutions of test substances were applied to sterile paper disks, dried until the solvent was completely removed, placed in a cup with the culture, and incubated for 2–4 days at 28 °C. To control fouling, cultures with disks were incubated for up to 7 days.

## 3. Results

**Figure 1:**
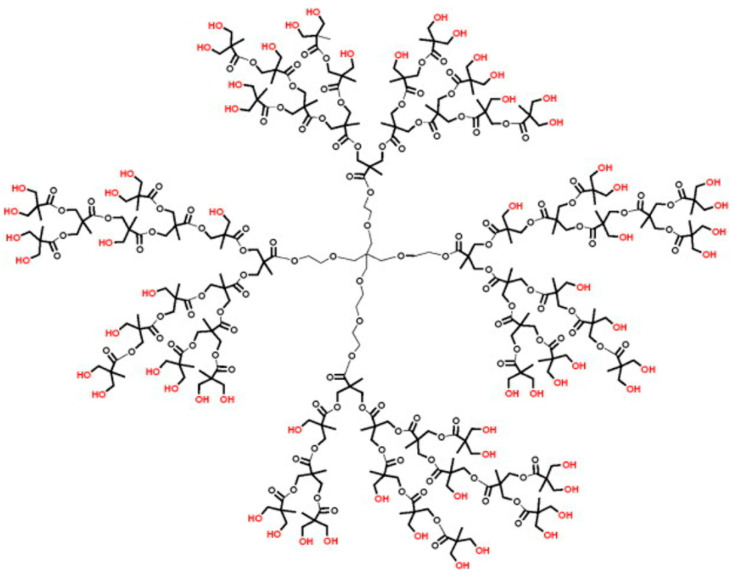
Structure of hyperbranched polyester polyol G4-OH.

The fourth-generation hyperbranched polyester polyol G4-OH (Figure 1) was chosen as a reductor and stabilizing agent for the synthesis of a CoNP nanocomposite. The presence of 64 hydroxyl groups in the G4-OH polyol makes it functionally active and can be used as a base platform for the coordination and stabilization of metal ions and nanoparticles. The terminal OH groups of the polyol in the system with the M^n+^ metal ion can be oxidized upon heating to form a metal–polymer nanocomposite in which metal nanoparticles will be immobilized in the hyperbranched polymer matrix.

In addition, the LD_50_ value ~2000 mg/kg indicates a low toxicity of the hyperbranched polyester polyol G4-OH, which creates the preconditions for the use of derivatives of this polymer for biomedical purposes [48].

### 3.1. Synthesis of Cobalt-Containing Nanocomposite CoNPs

The synthesis of the CoNP nanocomposite was performed by reducing cobalt chloride, CoCl_2_, in the hyperbranched polyester polyol G4-OH under heating in DMSO. Attempts were made to synthesize CoNPs in melting polyol. According to the experimental data, the color transitions correspond to the formation of polyol-stabilized cobalt nanoparticles, but the material is a viscous resin insoluble in water and traditional polar and nonpolar organic solvents in its aggregate state. Therefore, to obtain a colloid-resistant cobalt-containing nanocomposite, DMSO was used as an additional component to reduce the viscosity of the synthesis medium. The choice of DMSO is justified by the properties of a successful electrostatic stabilizer of cobalt nanoparticles [49]. It has been experimentally established that the best ratio of G4-OH:DMSO in the synthesis is 1:1.8 by weight.

**Figure 2:**
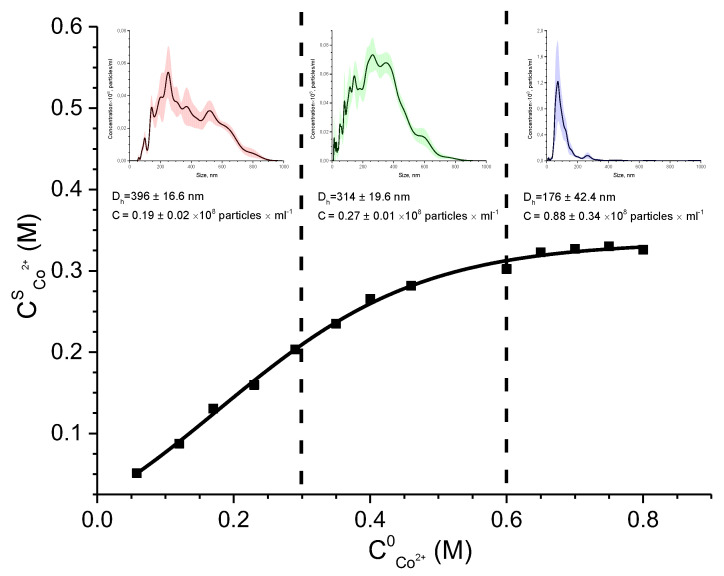
Distribution by size and number of particles in aqueous solution of G4-OH [Co^2+^-G4-OH], isotherms of sorption of Co^2+^ ions by G4-OH matrix in DMSO solution (C_G4-OH_ = 0.125 g·mL^−1^, C_Co2+_ = 0.05–0.8 M).

When developing the synthesis procedure, it was taken into account that the stage of interaction of metal ions with polyhydric alcohols determines the morphology and properties of metal nanoparticles in the polyol synthesis method. Data UV-Vis spectrophotometry (Figure S1) shows that the first stage of the reaction involves the immobilization of cobalt (II) ions in the polyol matrix with the formation of localized coordination units of tetrahedral geometry (d-d transition band at 530 nm with a shoulder at 490 nm). Sorption studies were carried out to estimate the minimum concentration of the polymer for the immobilization of cobalt ions at the stage of mixing and preorganization of reagents in the synthesis. To control the self-organization process in the [Co^2+^-G4-OH] system, an NTA analysis was carried out simultaneously. The sorption isotherm of Co^2+^ ions by the G4-OH matrix in DMSO solution and NTA analysis data are shown in Figure 2. 

It was established that when the G4-OH platform was saturated with Co^2+^ ions, the nanosystem was preorganized with the formation of monodisperse [*n*Co^2+^-*m*G4-OH] aggregates with a hydrodynamic diameter of 176 nm. The linearization of the sorption isotherm in the coordinates of the Langmuir model (Figure S2) was used to calculate the concentration constant of the sorption equilibrium K = 3.508 mL/mmol and the value of the limiting sorption of Co^2+^ A_∞_ = 3.204 mmol/g. According to the data of sorption studies at the preorganization stage in the [Co^2+^-G4-OH] system, theoretically, the ratio ν_Co(II)_/ν_OH polyeste rpolyol_ = 1:3 or 24 Co^2+^ ions per G4-OH molecule. But, in order for the polyol to act in the synthesis and as a reducing agent, it must be taken in large excess. Therefore, we decided to use the ratio ν_Co(II)_/ν_OH polyester polyol_ = 1:500 in the synthesis. 

By heating the reaction mixture of CoCl_2_ and G4-OH, cobalt-containing nanocomposite was produced for CoNPs-1 in the absence of NaOH and CoNPs-2 in the presence of NaOH. The reduction process of Co(II) by hyperbranched polyester polyol G4-OH was carried out by heating the reaction system in steps of 10 °C. The following color transitions were observed during the formation of CoNPs-1: blue (25 °C) to blue-green (170 °C) to dark green (210 °C) (Figure S3). The heating stage was 9 h. The brown color of the reaction mixture stabilized at 14 h. The introduction of NaOH into the reaction mixture increased the rate of deprotonation of the polyol [50] and accelerated the reaction for CoNPs-2. The color transitions were the same but at lower temperatures during the formation of CoNPs-2: blue at 25 °C, blue-green at 130 °C, and dark green at 140 °C (Figure S4). The heating stage was 7 h for CoNPs-2. The total synthesis time before the color stabilization of the reaction mixture was 14 h. Both CoNP samples, CoNPs-1 and CoNPs-2, were colloidally stable systems of light- and dark-brown color, respectively.

**Figure 3:**
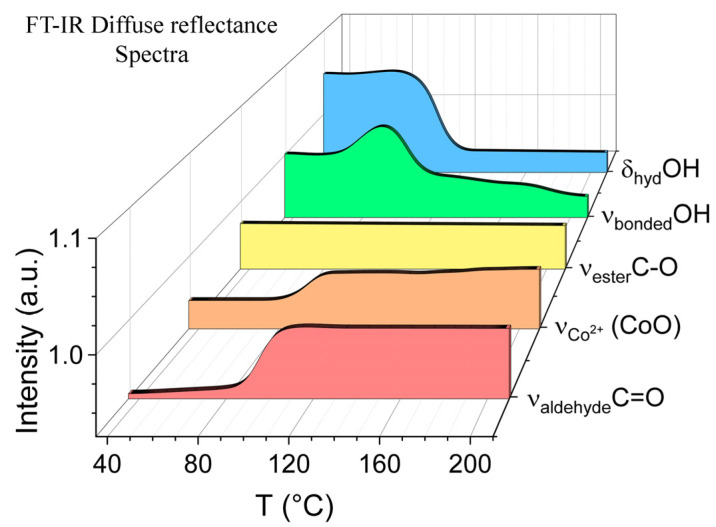
Profiles of intensity of characteristic bands in the FT-IR diffuse reflectance spectra of the [G4-OH-CoCl_2_] mixture depending on the heating (T = 35–210 °C, C_G4-OH_ = 0.629 g·mL^−1^, C_Co2+_ = 0.00258 g·mL^−1^).

The mechanism of synthesis of cobalt nanoparticles was studied using diffuse reflectance IR spectroscopy (Figure S5). Figure 3 shows the dynamic profiles of the intensity of the vibration bands of the characteristic groups OH_alcohol_, C=O_ester_, and C=O_aldehide_ depending on the heating temperature in situ. The process of oxidation of OH groups in G4-OH started from 90 °C. Oxidation of the alcohol groups of the hyperbranched polyester polyol occurred in the aldehyde groups. This was indicated by the appearance of and increase in the IR spectra of the band of stretching vibrations of the C=O bond in the aldehyde group at 1718 cm^−1^. The slight increase in the intensity of the signal of OH groups at 3439 cm^−1^ was associated with the formation of water molecules during the oxidation of the alcohol group, which then evaporated when the temperature rose to 100 °C. Above this temperature, a decrease in the intensity of the band of OH stretching vibrations of the alcohol groups of the polymer was observed, which indicates their oxidation. This effect was also confirmed by the decrease in the intensity of the band deformation vibrations of the OH groups of the polymer at 1314 cm^−1^. The correlation of the intensities of stretching and bending vibrations of the OH groups of the polyester polyol before and after the oxidation reaction showed that 6.1% of all OH groups of G4-OH were involved in the oxidation process. The appearance of a band at 667 cm^−1^ indicates the formation of a Co-O bond corresponds to the immobilization of cobalt nanoparticles in a hyperbranched polymer matrix. 

#### Studying the Stabilization of Cobalt Nanoparticles 

**Figure 4:**
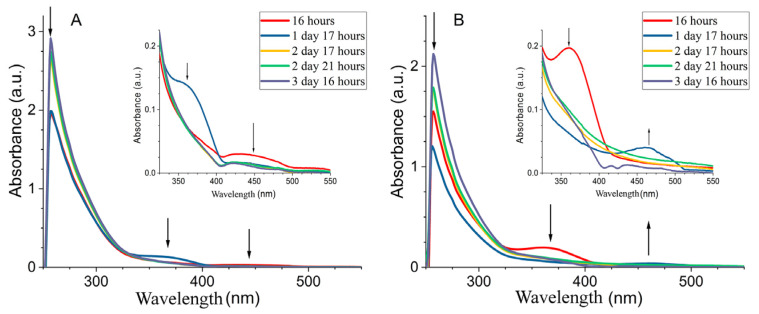
Evolution of absorption spectra in the CoNPs-1 system (**A**) CoNPs-2 (**B**) in situ.

The in situ stabilization of cobalt nanoparticles in metal–polymer nanocomposite CoNPs was studied using UV-Vis spectroscopy (Figure 4). The surface plasmon resonance (SPR) bands of the oxide-phase Co_x_O_y_ [51] and metallic cobalt particles Co^0^ [52] were located at 250 nm and 403 nm, respectively, in the absorption spectra of first-day-produced CoNPs. The reverse and direct Ostwald mechanisms for the formation of cobalt nanoparticles in the polymer matrix for CoNPs-1 and CoNPs-2 are shown by the in situ development of SPR in the CoNP dispersions, respectively. The CoNP nanocomposites required a total of 4 days for the cobalt metal nanophase to stabilize. 

**Figure 5:**
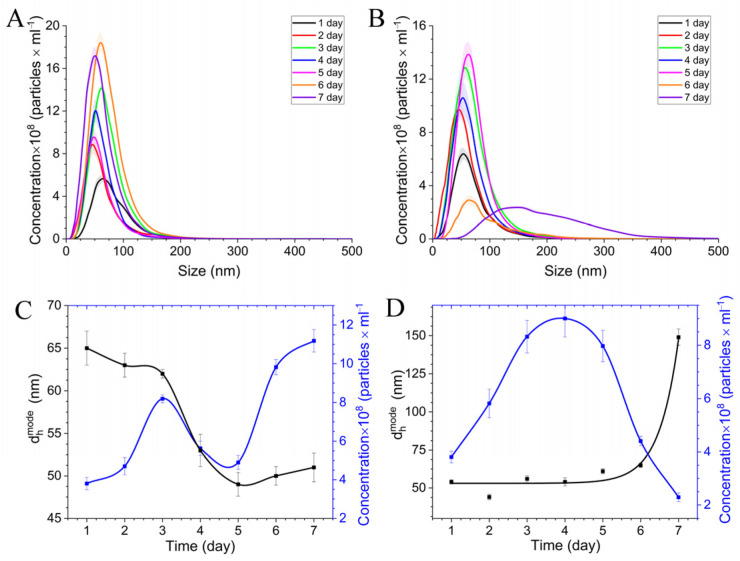
NTA data of nanocomposite dispersions CoNPs-1 (**A**) and CoNPs-2 (**B**) in the DMSO; profiles of the hydrodynamic diameter and concentration of particles in colloidal solutions of CoNPs-1 (**C**) and CoNPs-2 (**D**) samples in situ.

Additionally, size, i.e., the hydrodynamic diameter of the CoNP particles (d_h_) during in situ stabilization, was controlled using the NTA method (Figure 5). NTA kinetic studies of dispersions of cobalt-containing nanocomposites in situ for 7 days indicate the monodisperse aggregates were formed during stabilization in a DMSO solution (Figure 5A,B).

A two-dimensional analysis of NTA data from the stabilization time of the nanocomposite CoNPs by the hydrodynamic diameter of the main fraction (d_h_^mode^) and particle concentration (Figure 5C,D) confirm the spectrophotometrically established stabilization mechanisms. For CoNPs-1, stabilization by the reverse Ostwald mechanism was accompanied by a decrease in hydrodynamic diameter with increasing particle concentration (Figure 5C). For CoNPs-2, inverse dependences of the hydrodynamic diameter and increasing particle concentration (Figure 5D) were observed, which indicate stabilization by the direct Ostwald mechanism. The cobalt nanophase stabilization process ended with the formation of monodisperse aggregates of colloidally stable nanocomposites with a hydrodynamic diameter of 51 nm for CoNPs-1 and 147 nm for CoNPs-2.

### 3.2. Spectral Properties of Nanocomposite CoNPs

**Figure 6:**
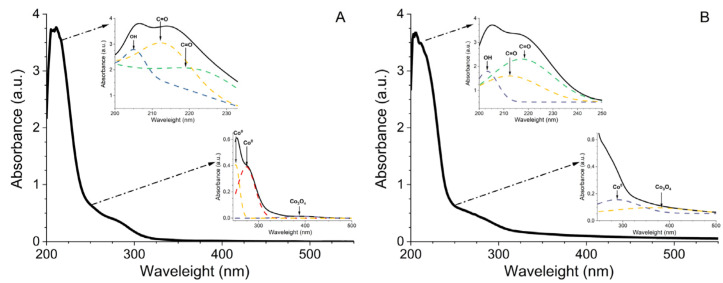
Electronic absorption spectra of dispersions of individual polymer-stabilized CoNPs-1 (**A**); CoNPs-2 (**B**) nanoparticles in methanol–water mixture (1:1) (C_CoNPs_ = 0.02 g/mL, the dotted lines are interpolation of absorption band positions from Gauss distribution function R^2^ = 99.9, χ^2^ = 3.97 × 10^−5^).

The produced colloidally stable cobalt-containing nanocomposite CoNPs’ spectrum properties were investigated. Data on the cobalt nanophase were gathered using UV-Vis spectroscopy. The Gaussian distribution function was used to interpolate the locations of the absorption bands in order to investigate the electronic absorption spectra of CoNP dispersions in the methanol–water (1:1) system (Figure 6). Low-dimensional clusters (SPR = 250 nm) and metallic cobalt nanoparticles (SPR = 273 nm) [53] as well as the oxide-phase Co_3_O_4_ (SPR = 396 nm) were used to simulate the cobalt nanophase in CoNPs-1. Metallic Co^0^ nanoparticles with an SPR of 276 nm and cobalt oxides with an SPR of 390 nm were used to simulate CoNPs-2 particles. The n-π electron transitions in the distinctive groups of the stabilizer G4-OH were the subject of the absorption bands between 200 and 230 nm. 

**Figure 7:**
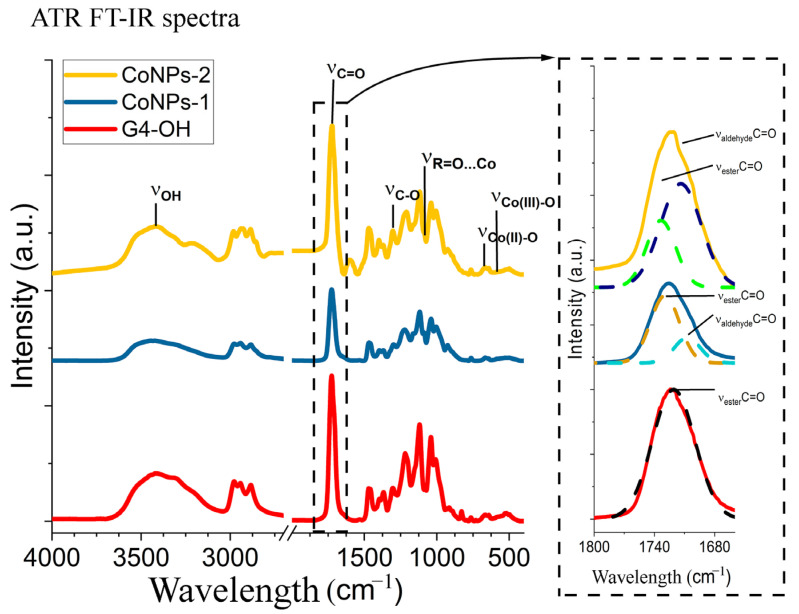
FT-IR spectra and the region of ester bonds with deconvolution for the initial and obtained compounds.

The localization sites of CoNPs in the G4-OH polymer matrix were evaluated using FTIR spectroscopy (Figure 7). For both samples of CoNPs, the FTIR spectra are characterized by the presence of absorption bands at 668 cm^−1^ and 579 cm^−1^ due to the valence vibrations of ν_(Co(II)-O)_ and ν_(Co(III)-O)_ in cobalt oxide [53]. The band of valent vibrations of the Co...O-H bond at 1090 cm^−1^ indicates the localization of cobalt nanoparticles due to nonreducing peripheral hydroxyl groups of polyol. This effect was confirmed by a decrease in the intensity of the corresponding OH group oscillation bands of polyol G4-OH at 1298, 1048, and 1008 cm^−1^. The band at 1730 cm^−1^ decreased during synthesis. This indicates that the metal phase’s localization of the also occurred with the participation of carbonyl groups of polyester polyol G4-OH.

### 3.3. Morphology of Nanocomposite CoNPs

The concentration of cobalt in the obtained nanocomposite CoNPs was determined using complexometric titration. According to the titration dates, the concentration of cobalt was 0.13 mg·g^−1^ and 0.24 mg·g^−1^ for CoNPs-1 and CoNPs-2, respectively.

**Figure 8:**
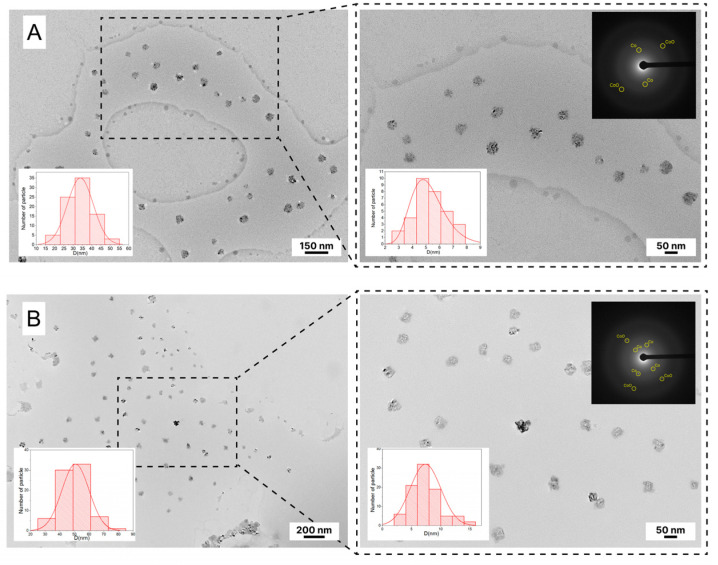
TEM images of CoNPs-1 (**A**) and CoNPs-2 (**B**), with inset size distribution and SAED diffraction.

Transmission electron microscopy (TEM) and electron diffraction were used to assess the morphology of the produced CoNPs (Figure 8). According to TEM, both samples of CoNPs formed nanoparticles with spheroidal structures. A polymer aggregate with a diameter of 35 ± 10 nm with immobilized cobalt nanoparticles with a diameter of 5 ± 2 nm is what makes up the CoNPs-1 nanocomposite. The CoNPs-2 nanocomposite has larger spheroid particles. The polymer aggregate particles have a diameter of 50 ± 10 nm, and the size of immobilized cobalt nanoparticles is also larger and equal to 7 ± 3 nm. The estimated interplanar distance (d) values show that both samples include the metallic phases Co^0^ and CO_3_O_4_ (Fm3m-face-centered cubic crystal lattice).

**Figure 9:**
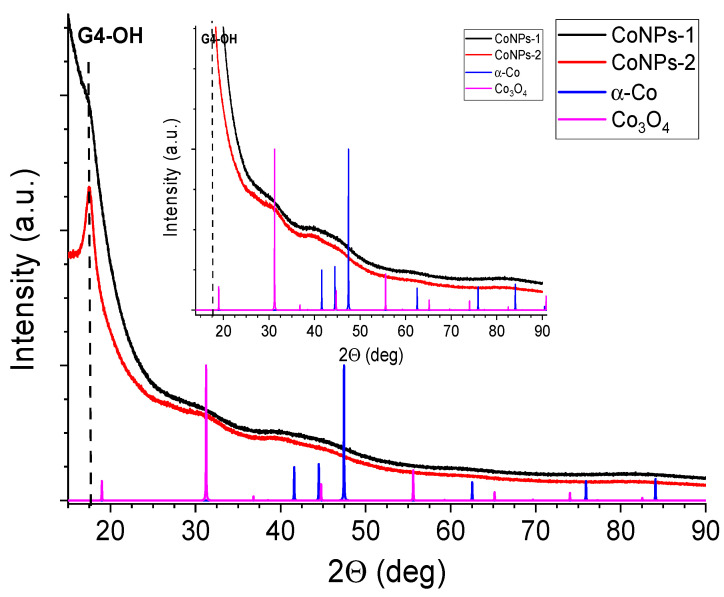
XRD spectra of CoNPs-1 and CoNPs-2.

By using X-ray phase analysis, the morphology of the produced CoNP nanocomposites was assessed. Qualitative analysis shows that both samples of CoNP cobalt nanoparticles contained a polymer matrix reflex at 2θ equal to 17.69°. X-ray phase analysis showed the presence of α-Co^0^ metallic nanoparticles in the samples, the corresponding signals were observed at 2θ angles equal to 41.48° and 62.55° for both CoNP samples (Figure 9). In addition, both CoNP samples contained the Co_3_O_4_ oxide phase characterized by reflexes at 31.25° and 44.94°.

### 3.4. Thermal Stability of Nanocomposite CoNPs

Data TG analysis (Figures S6–S8) of G4-OH and the synthesized CoNPs showed that the nanocomposites were stable in the temperature range of 25–260 °C. Endo-effects at 100 °C are associated with the removal of H_2_O and DMSO molecules from the cavities of hyperbranched polyester polyol. The thermolysis temperature of G4-OH, CoNPs-1, and CoNPs-2 was 359.7, 359.2, and 372.6 °C, respectively. This indicates an alloying effect with an increase in the content of immobilized cobalt nanoparticles in the nanocomposite. However, too low a load of cobalt nanoparticles did not allow us to estimate their content from the TG curves.

### 3.5. Magnetic Properties of Nanocomposite CoNPs

**Figure 10:**
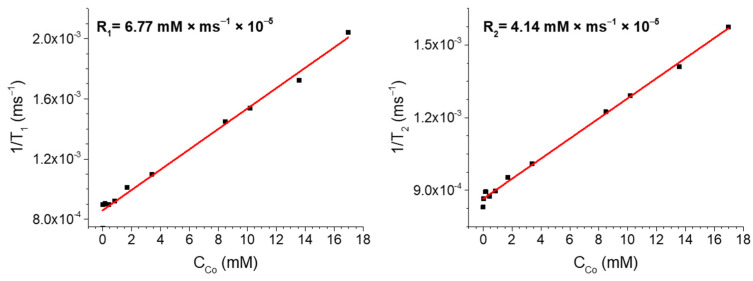
Reciprocal *T*_2_ and *T*_1_ vs. CoNPs-2 concentrations in DMSO (65%).

MRI is currently a convenient diagnostic method that allows obtaining detailed images of human internal organs and tissues without the use of X-rays. One way to improve image quality in MRI is through the use of nanoparticles. At the same time, nanoparticles must have certain properties: magnetic sensitivity and biocompatibility. Therefore, the magnetic relaxation parameters of the obtained nanocomposite CoNPs were studied using the method of NMR relaxation. We failed to obtain good relaxation data for the CoNPs-1 nanocomposite, since the concentration of magnetically active cobalt nanoparticles is very low. Studies of relaxation properties were successful only for CoNPs-2, with a metal content 2 times higher. The dependence of the magnetic relaxation parameters of CoNPs-2 in the concentration range of 0.017–17 mM (1–1000 µg/mL) was studied (Figure 10).

Relaxometric measurements show that the transverse and longitudinal relaxations were 6.77 mM·ms^−1^ × 10^−5^ and 4.14 mM·ms^−1^ × 10^−5^, for R_2_ and R_1_, respectively. The low R_2_/R_1_ = 0.61 ratio [54] indicates that the synthesized nanocomposite CoNPs-2 are rather *T*_1_ contrast agents. The linear dependence of the change in relaxivity over the entire concentration range confirms the individual composition and the absence of aggregation between nanoparticles in solution, which correlates with the polydispersity data obtained using NTA (Figure 5). In addition, the linear nature of the change in relaxivity shows the same ratio of the oxide shell and ZVcore for all particles, which statistically confirms the data obtained using TEM (Figure 8).

### 3.6. Biological Properties

#### 3.6.1. Hemolytic Activity of Nanocomposite CoNPs

**Figure 11:**
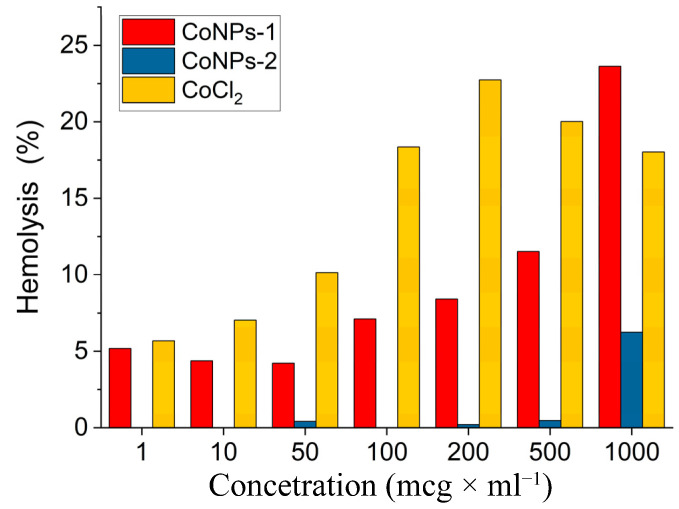
Effect of CoNP concentration on hemolysis (C_CoNPs_ = 1–1000 mcg·mL^−1^).

Hemolytic activity was assessed using the Klajnert method [47]. The study was performed on blood containing 3% sodium citrate taken from the ulnar vein of a healthy donor, diluted with PBS in a ratio of 1:1. Substances with hemolytic activity less than 10% are not potentially dangerous to the body and can be used for further developments in biomedicine and pharmacology. The hemolytic activity (in%) of salt CoCl_2_, CoNPs-1, and CoNPs-2 in the concentration range of 1–1000 µg/mL was evaluated (Figure 11). Nanocomposites CoNPs-1 and CoNPs-2 were found to have low hemolytic activity, which allows the positioning of these for further biomedical developments. The value of HC_50_ for CoCl_2_ is 2.82 g/mL, for CoNPs-1 is 3.17 g/mL and for CoNPs-2 is 8.89 g/mL.

#### 3.6.2. Antiproteinase Activity of Nanocomposite CoNPs

**Figure 12:**
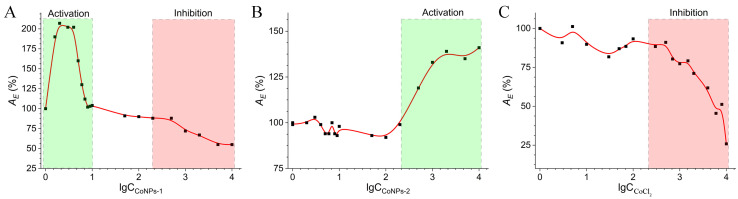
Enzymatic activity (*A_E_*, %) of chymosin in the presence of nanocomposites CoNPs (**A**,**B**) and CoCl_2_ (**C**).

The modulator activity of the synthesized CoNP samples was studied. The enzymatic activity of chymosin in the presence of salt CoCl_2_, hyperbranched polyester polyol G4-OH, and synthesized CoNP nanocomposites was evaluated (Figure 12). It was established that polyester polyol G4-OH is not a modulator of chymosin activity. 

A comparative analysis of the enzymatic activity of chymosin in the presence of CoCl_2_ and of synthesized samples of CoNPs is represented in Table 1. It was found that the CoCl_2_ exhibited an inhibitory effect with increasing concentration. It was established that the CoNPs-1 nanocomposite with a reduced cobalt content had both an activating and inhibitory effect on the enzyme activity, depending on the concentration range. At the same time, the nanocomposite CoNPs-2 with a higher metal load was only an activator.

To assess the value of half inhibition (IC_50_) and half activation (AC_50_), the dependences of the enzymatic activity of chymosin in the presence of modulators (CoCl_2_, CoNPs) were evaluated in individual ranges of inhibition and activation concentrations (Table 1). Thus, the synthesized CoNPs nanoparticles can be used to control the activity of proteolytic enzymes.

#### 3.6.3. Antimycotic Activity of Nanocomposite CoNPs

The antimycotic activity of the G4-OH polyol and CoNP nanocomposites was studied using a disk diffusion test. *Candida albicans* and *Aspergillus niger* were chosen as fungal cultures for the study as the most pathogenic and resistant. For research, samples of polymer and nanocomposites were used at a concentration of 2 µg/mL, which corresponds to the allowable concentration for biomedical use. Unfortunately, for the CoNPs-1 nanocomposite, it was not possible to reveal antimycotic properties at the studied concentration. It is possible that the antimycotic effect would manifest itself in a higher concentration that is acceptable for disinfectants. It was found that the synthesized CoNPs-2 composite had antimycotic activity towards *Aspergillus fumigatus* F-753 (Table 2, Figure S9).

## 4. Conclusions

Thus, a methodology for the hyperbranched polyol process for obtaining colloidal-cobalt-containing nanocomposite CoNPs was developed. It was proven that hyperbranched polyester polyols can be not only successful stabilizers of metal nanoparticles, but also excellent reducing agents. Studies of the reducing properties of hyperbranched polyols will be continued to find the optimal conditions for their action and determine approaches to control the morphology of composite nanomaterials in a hyperbranched polyol process. The synthesized composite nanomaterial CoNPs is a colloidally stable spheroidal aggregates consisting of metal nanoparticles with a core (Co^0^)–shell (Co_3_O_4_) structure immobilized in a matrix of the fourth-generation hyperbranched polyester polyol G40-OH. 

The hemolytic activity of the synthesized CoNPs nanocomposites is significantly lower than that of the inorganic salt CoCl_2_, which makes it possible to position them as advanced nontoxic reagents for biotechnology, pharmacy, medicine, and theranostics. Based on the multifunctionality of the CoNPs nanocomposite, several directions for further practical developments can be singled out at once. First, the synthesized nanoparticles can be studied as *T*_1_ contrast agents for MRI diagnostics. Second, optimization of the composition of nanocomposites and control of the loading and morphology of the metal phase can open the way to the creation of nontoxic effective enzyme modulators and antibacterial reagents with high colloidal stability and biocompatibility. Thirdly, the proposed approach can become universal for the one-stage synthesis of colloidally stable metal–polymer nanocomposites containing nanoparticles of transition and noble metals in a two-component mixture of metal salt–hyperbranched polyol.

## Figures and Tables

**Table 1:** Enzymatic activity of CoNPs towards chymosin (C_CoNPs_ = 1–10,000 µg/mL).

Sample	*A_E_*, %	Effect	C_CoNPs_, μg/mL	AC_50_, mg/mL	IC_50_, mg/mL
CoNPs-1	210 ± 5	Activation	0.1–2	0.0047	-
	54 ± 3	Inhibition	5000–10,000	-	10
CoNPs-2	134 ± 5	Activation	500–10,000	12.8	-
CoCl_2_	53 ± 8	Inhibition	400–10,000	-	0.6

**Table 2:** Antimycotic activity of G4-OH and nanocomposite CoNPs (C_CoNPs_ = 2 µg/mL).

Sample	Zone of Inhibition (mm) (Figure S10)	
	*Candida albicans* ATCC 10201	*Aspergillus fumigatus* F-753
G4-OH	0	0
CoNPs-2	13.6	25.2
Nystatin	15.8	13.5
Ketoconazole	34.7	11.3

## Data Availability

Not applicable.

## Supplementary Materials

The following supporting information can be downloaded at: https://www.mdpi.com/article/10.3390/polym15153248/s1. Figure S1. Electronic absorption spectra of solutions of Co(II) ions before and after binding with polyester polyol G4-OH in DMSO (C_G4-OH_ = 0.125 g·mL^−1^, C_Co(II)_ = 0.1 M, I = 0.1 M LiClO_4_); Figure S2. Isotherm of sorption of Co^2+^ by G4-OH matrix in Langmuir coordinates (R^2^ = 1, χ^2^ = 2.01049 × 10^−29^); Figure S3. Change in the color of the solution during the synthesis of CoNPs-1 cobalt nanoparticles: (a) 25 °C, (b) 170 °C, (c) 210 °C, and (d) 14 h after synthesis; Figure S4. Change in the color of the solution during the synthesis of CoNPs-2 cobalt nanoparticles: (a) 25 °C, (b) 130 °C, (c) 140 °C, and (d) 14 h after synthesis; Figure S5. FT-IR spectra of diffuse reflectance of [G4-OH-CoCl_2_] mixture in situ when heated (T = 35–210 °C, C_G4-OH_ = 0.629 g·mL^−1^, C_Co2+_ = 0.00258 g·mL^−1^); Figure S6. TG-DSC curves of G4-OH; in argon atmosphere (Pt-Rh crucible 10 K·min^−1^); Figure S7. TG-DSC curves of CoNPs-1; in argon atmosphere (Pt-Rh crucible 10 K·min^−1^); Figure S8. TG-DSC curves of CoNPs-2; in argon atmosphere (Pt-Rh crucible 10 K·min^−1^); Figure S9. NMR relaxation of CoNPs-2 from concentration in DMSO/H2O solution; Figure S10. Zone of inhibition of cell growth of *Candida albicans* (A) and *Aspergillus fumigatus* (B) A1—CoNP-2, A2—DMSO, A3—[G4-OH-DMSO], A4—nystatin, A5—ketoconazole.
