# Supplementary material for: A Hyperbranched Polyol Process for Designing and Manufacturing Nontoxic Cobalt Nanocomposite
Source: Polymers (Basel). 2023 Jul 30;15(15):3248. doi: 10.3390/polym15153248 (PMC10421248; doi:10.3390/polym15153248)
Supplement: Supplementary file 1 [file polymers-15-03248-s001.zip › polymers-2505707-supplementary.pdf]

A Hyperbranched Polyol Process for Designing and Manufacturing Nontoxic Cobalt Nanocomposite

Anastasia Burmatova [1], Artur Khannanov [1,*], Alexander Gerasimov [1], Klara Ignateva [1], Elena Khaldeeva [1,2], Arina Gorovaia [1], Airat Kiiamov [3], Vladimir Evtugyn [1] and Marianna Kutyreva [1]

[1] M. Butlerov Chemical Institute, Kazan Federal University, 18 Kremlyovskaya Str., 420008 Kazan, Russia; nastyaburmatova15@gmail.com (A.B.); alexander.gerasimov@kpfu.ru (A.G.); toklara@yandex.ru (K.I.); e_khaldeeva@mail.ru (E.K.); gorovayaarina5@gmail.com (A.G.); vevtugyn@gmail.com (V.E.); mkutyreva@mail.ru (M.K.)

[2] Kazan Research Institute of Epidemiology and Microbiology, 67 Bolshaya Krasnaya Str., 420015 Kazan, Russia

[3] Quantum Simulators Lab, Institute of Physics, Kazan Federal University, Kremlevskaya Str. 18, 420008 Kazan, Russia; airatphd@gmail.com

* Correspondence: aahannanov@kpfu.ru

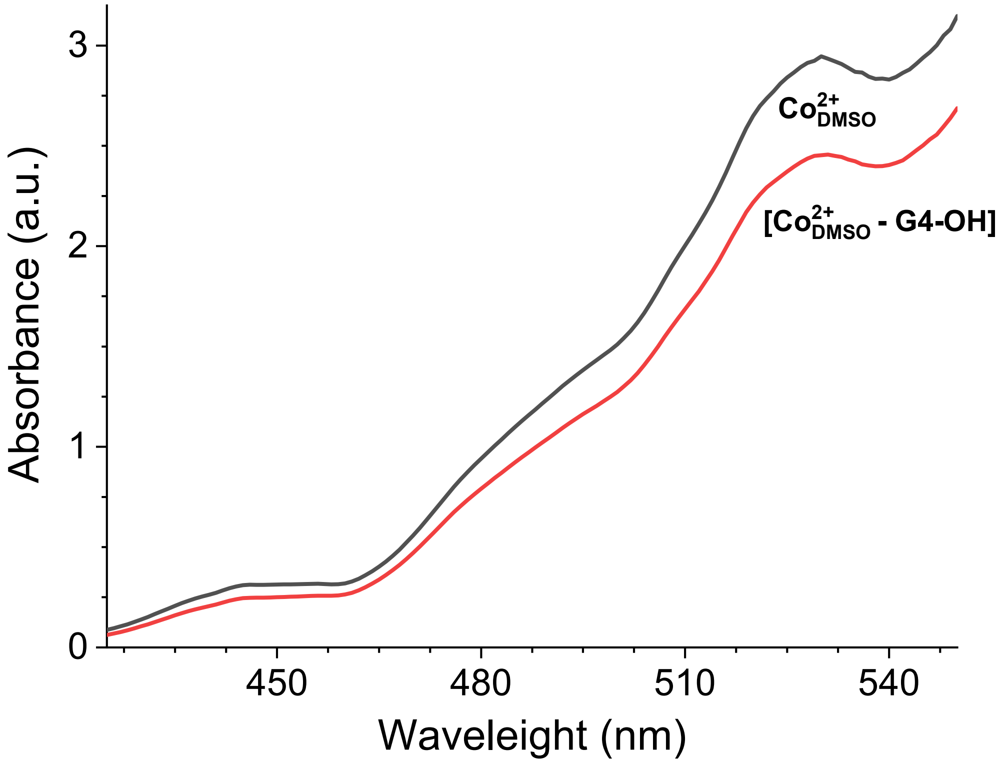

Figure S1. Electronic absorption spectra of solutions of Co(II) ions before and after binding with polyester polyol **G4-OH** in DMSO ($C_{G4\text{-}OH} = 0.125$ g × ml^{-1}, $C_{Co(II)}$=0.1 M, I=0.1 M $LiClO_4$).

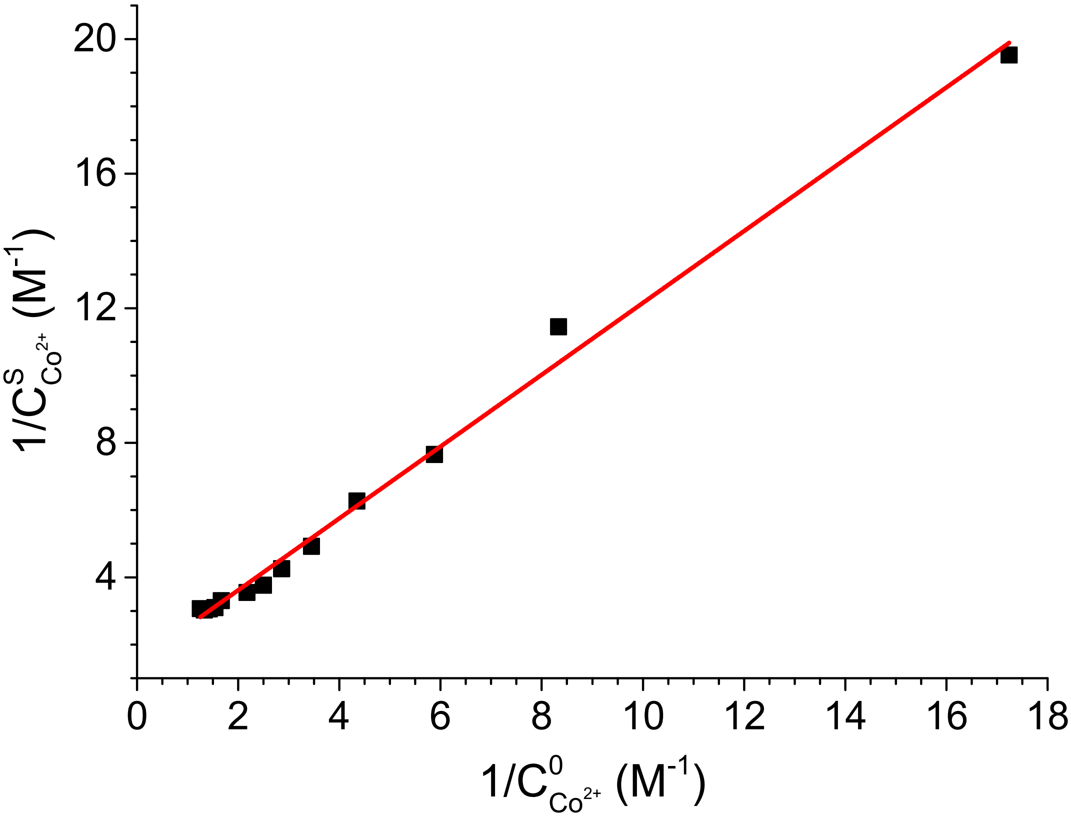

Figure S2. Isotherm of sorption of Co^{2+} by G4-OH matrix in Langmuir coordinates (R^2=1, χ^2=2.01049 × 10^{-29}).

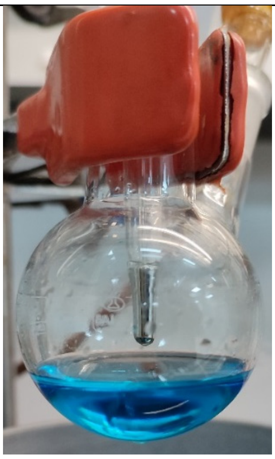	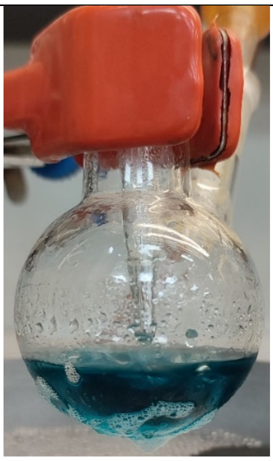	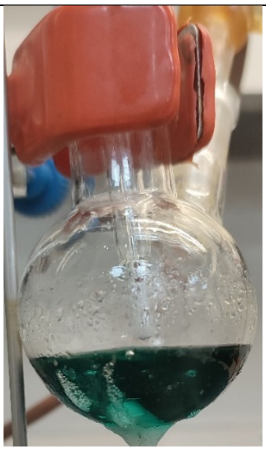	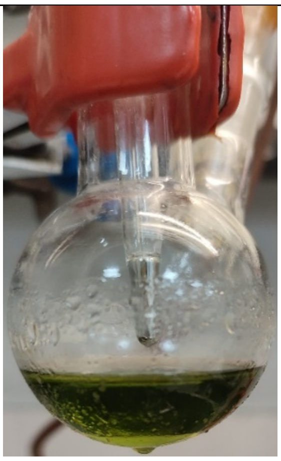
(a) Initial synthetic solution	(b) synthetic solution at 170 °C	(c) synthetic solution at 170 °C	(d) synthetic solution at 25 °C, after synthesis

Figure S3. Change in the color of the solution during the synthesis of **CoNPs-1** cobalt nanoparticles: (a) 25 °C, (b) 170 °C, (c) 210 °C, (d) 14 hours after synthesis.

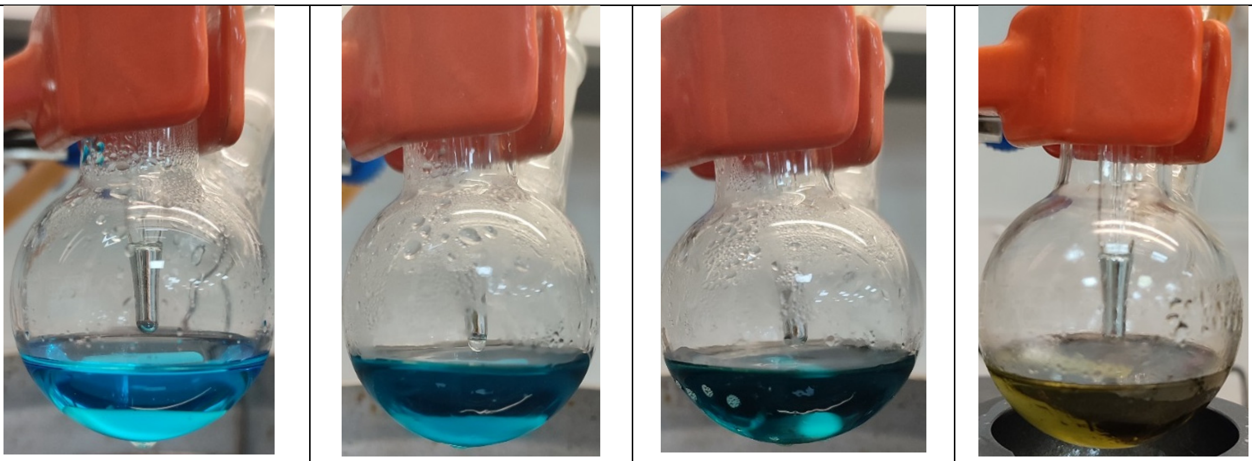

(a) Initial synthetic solution	(b) synthetic solution at 130 °C	(c) synthetic solution at 140 °C	(d) synthetic solution at 25 °C, after synthesis

Figure S4. Change in the color of the solution during the synthesis of **CoNPs-2** cobalt nanoparticles: (a) 25 °C, (b) 130 °C, (c) 140 °C, (d) 14 hours after synthesis.

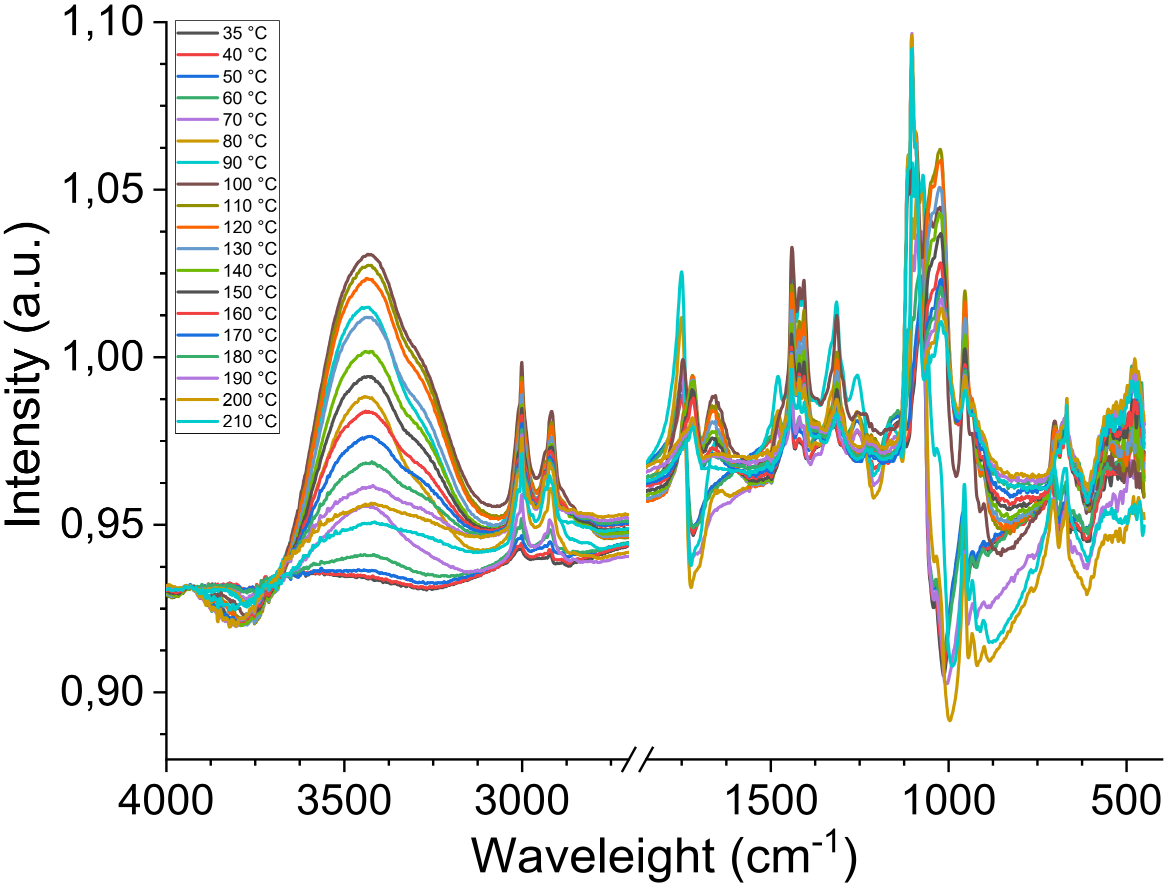

Figure S5. FT-IR spectra of diffuse reflectance of [**G4-OH** - $CoCl_2$] mixture in situ when heated (T=35-210 °C, $C_{G4\text{-}OH}$ = 0.629 g × ml^{-1}, C_{Co2+} = 0.00258 g × ml^{-1}).

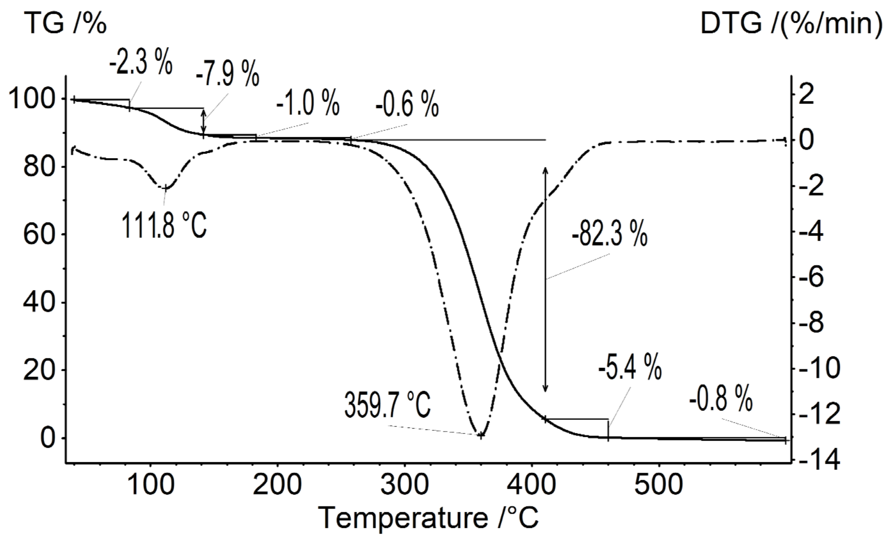

Figure S6. TG- DSC curves of **G4-OH**; in argon atmosphere (Pt-Rh crucible 10 K × min^{-1}).

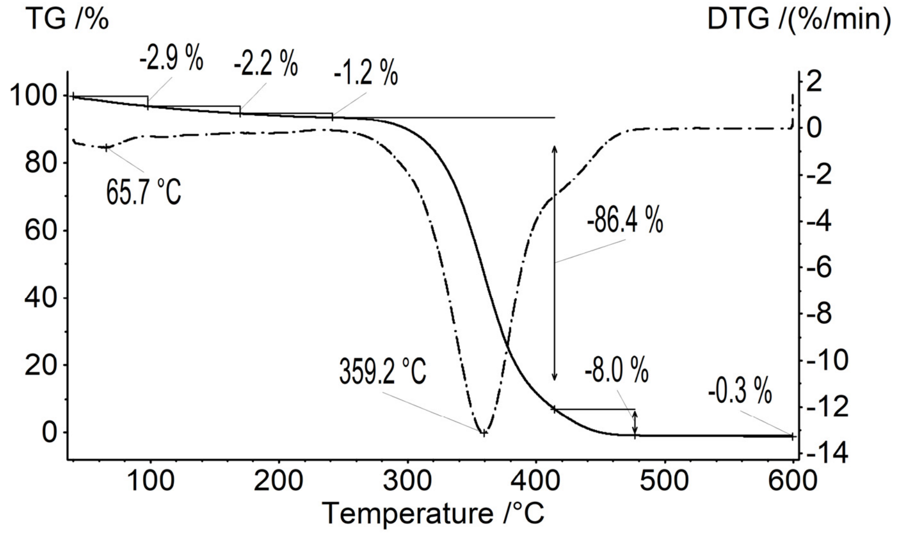

Figure S7. TG- DSC curves of **CoNPs-1**; in argon atmosphere (Pt-Rh crucible 10 K × min^{-1}).

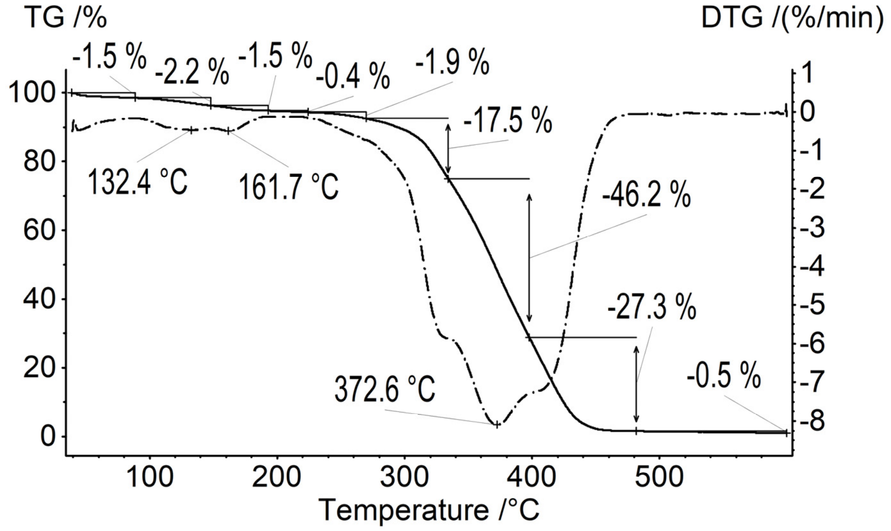

Figure S8. TG-DSC curves of **CoNPs-2**; in argon atmosphere (Pt-Rh crucible 10 K × min^{-1}).

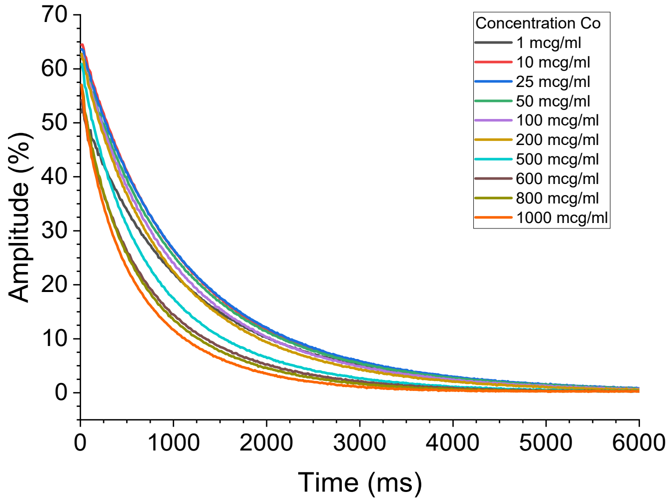

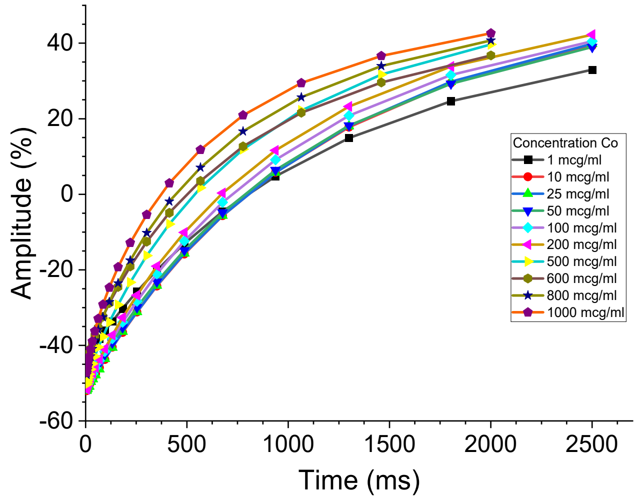

Figure S9. NMR relaxation of CoNPs-2 from concentration in DMSO/H2O solution.

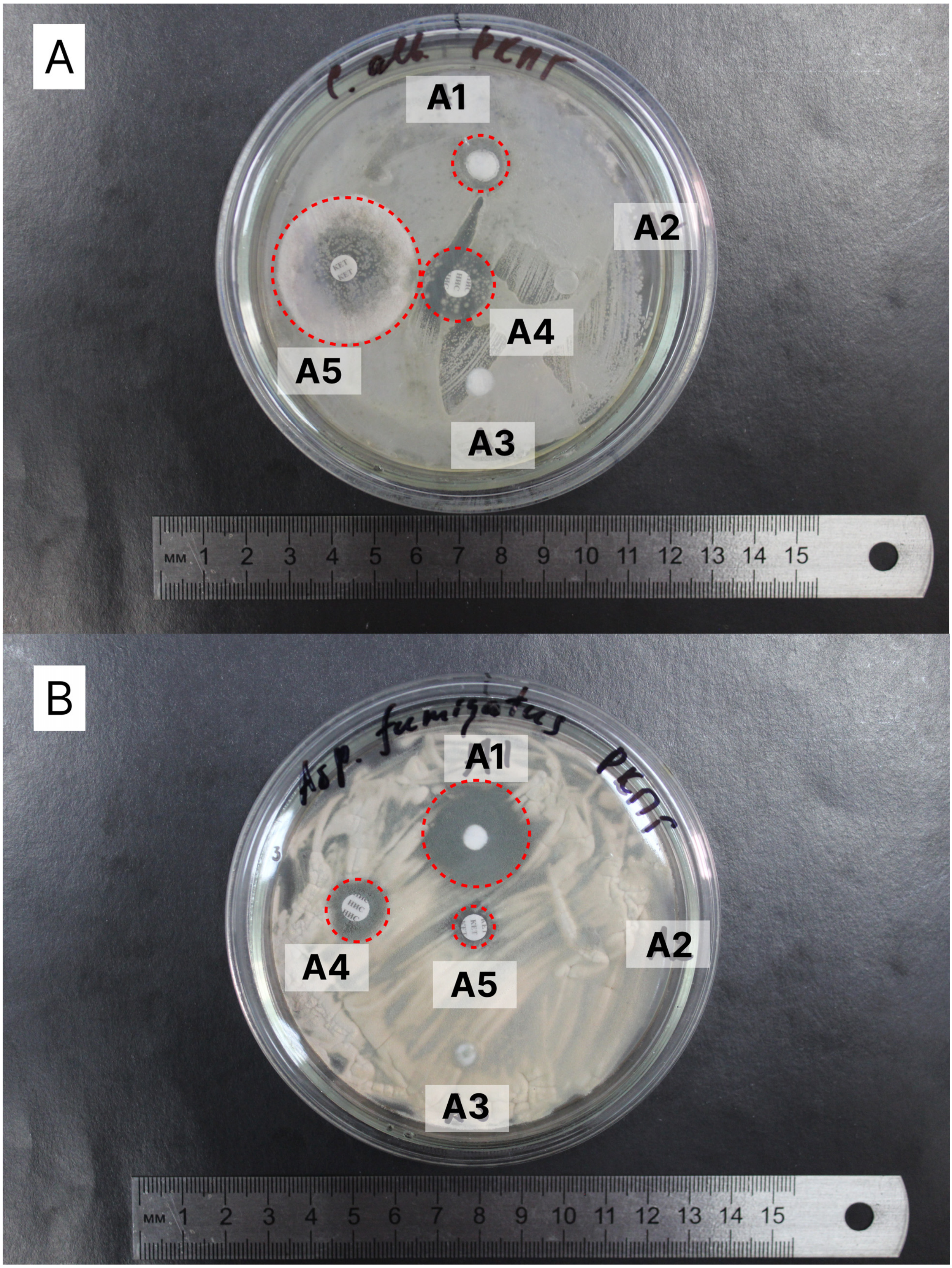

Figure S10. Zone of inhibition of cell growth of *Candida albicans* (A) and *Aspergillus fumigatus* (B): A1 - **CoNP-2**, A2 - DMSO, A3 - [**G4-OH**-DMSO], A4 - Nystatin, A5 - Ketoconazole
